# Sodium-Dependent Phosphate Transporters in Osteoclast Differentiation and Function

**DOI:** 10.1371/journal.pone.0125104

**Published:** 2015-04-24

**Authors:** Giuseppe Albano, Matthias Moor, Silvia Dolder, Mark Siegrist, Carsten A. Wagner, Jürg Biber, Nati Hernando, Willy Hofstetter, Olivier Bonny, Daniel G. Fuster

**Affiliations:** 1 Division of Nephrology, Hypertension and Clinical Pharmacology, University Hospital of Bern, Bern, Switzerland; 2 Institute of Biochemistry and Molecular Medicine, University of Bern, Bern, Switzerland; 3 NCCR Transcure, University of Bern, Bern, Switzerland; 4 Department of Pharmacology and Toxicology, University of Lausanne, Lausanne, Switzerland; 5 Group for Bone Biology and Orthopaedic Research, Department of Clinical Research, University of Bern, Bern, Switzerland; 6 Institute of Physiology, University of Zürich, Zürich, Switzerland; 7 NCCR Kidney.CH, University of Zürich, Zürich, Switzerland; Faculté de médecine de Nantes, FRANCE

## Abstract

Osteoclasts are multinucleated bone degrading cells. Phosphate is an important constituent of mineralized bone and released in significant quantities during bone resorption. Molecular contributors to phosphate transport during the resorptive activity of osteoclasts have been controversially discussed. This study aimed at deciphering the role of sodium-dependent phosphate transporters during osteoclast differentiation and bone resorption. Our studies reveal RANKL-induced differential expression of sodium-dependent phosphate transport protein IIa (NaPi-IIa) transcript and protein during osteoclast development, but no expression of the closely related NaPi-IIb and NaPi-IIc SLC34 family isoforms. *In vitro* studies employing NaPi-IIa-deficient osteoclast precursors and mature osteoclasts reveal that NaPi-IIa is dispensable for bone resorption and osteoclast differentiation. These results are supported by the analysis of structural bone parameters by high-resolution microcomputed tomography that yielded no differences between adult NaPi-IIa WT and KO mice. By contrast, both type III sodium-dependent phosphate transporters Pit-1 and Pit-2 were abundantly expressed throughout osteoclast differentiation, indicating that they are the relevant sodium-dependent phosphate transporters in osteoclasts and osteoclast precursors. We conclude that phosphate transporters of the SLC34 family have no role in osteoclast differentiation and function and propose that Pit-dependent phosphate transport could be pivotal for bone resorption and should be addressed in further studies.

## Introduction

Phosphate is a major constituent of hydroxyapatite (Ca_3_(PO4)_2_Ca(OH)_2_) and thus of mineralized bone. Bone matrix synthesis and mineralization, i.e. hydroxyapatite deposition, is controlled by osteoblasts. The reverse process (bone resorption) is achieved by multinucleated osteoclasts. Osteoclasts are polarized cells with a basolateral side facing the blood compartment and an apical side characterized by a ruffled border facing bone matrix and delineating a resorption lacuna. Tight seals isolate the basolateral from the apical compartment, allowing creation of a dedicated apical environment. The low pH in the resorption lacuna is generated by active H^+^- pumping of the V-ATPase residing on the ruffled membrane [1]. The high H^+^ concentration is critical for the optimal catalytic activity of bone matrix degrading enzymes and supports the dissolution of hydroxyapatite [2]. The latter process leads to the liberation of large amounts of calcium and phosphate in the hemivacuole, where concentrations of up to 40 mM calcium can be reached [3]. Thus, during active bone resorption, osteoclasts are exposed to high ambient concentrations of calcium and phosphate, especially at the apical, resorbing site. Three putative exit pathways from the hemivacuole can be envisioned including transcytosis, leakage at the sealing zone or transcellular transport involving membrane transporters and/or channels at the apical and basolateral membranes [4]. While transcytosis seems to be the main export pathway for matrix collagens [5,6], experimental evidence favors the transcellular transport in the case of calcium [4,7]. How phosphate exits the resorption lacuna is currently not clear, but both sodium-dependent and sodium-independent phosphate transport systems have been described in osteoclasts that may facilitate transcellular phosphate transport in the osteoclast [8–13]. The sodium-independent, acid-activated phosphate transport system in osteoclasts has been characterized functionally in great detail but its molecular identity remains enigmatic [12,13]. In contrast, more information is available on the molecular nature of the sodium-dependent phosphate transport system. Of the three types of sodium-dependent phosphate transporter classes recognized in mammals (types I (SLC17 family), II (SLC34 family) and III (SLC20 family)), type II and type III phosphate transporters were previously identified in osteoclasts [8,12]. While there is general agreement on the expression of the two type III phosphate transporters Pit-1 and Pit-2, discrepant findings were reported with regards to the NaPi-IIa isoform in osteoclasts. Gupta et al. demonstrated on RNA and protein level the expression of NaPi-IIa in osteoclasts from several species, including mouse, chicken and rabbit [8–11]. The NaPi-IIa mRNA identified in osteoclasts was identical to the one in the kidney and not an isoform or splice variant thereof [8]. By immunofluorescence staining, NaPi-IIa localized to the basolateral membrane of osteoclasts, at the opposite site of the ruffled border [11]. In contrast, Ito et al. were unable to detect NaPi-IIa protein expression in osteoclasts by Western blot or immunofluorescence [12].

Bone histology analysis of full body NaPi-IIa KO mice revealed poorly developed metaphyseal trabeculae and retarded secondary ossification at 4 weeks of age. At 16 weeks of age, however, the number of metaphyseal trabeculae in KO mice was found to be significantly greater than that of WT littermates [14]. In another study, no differences in histomorphometric bone parameters were found between 12 week old NaPi-IIa KO and WT mice, but there was a non-significant trend towards increased bone volume in the KO mice [15]. These data indicate that there is an age-dependent adaptation in the bone upon loss of NaPi-IIa in mice.

NaPi-IIa is a key phosphate transporter in the kidney. The full body NaPi-IIa KO mice exhibit hypophosphatemia with hyperphosphaturia, reduced PTH, elevated 1,25-OH vitamin D_3_ levels with secondary hypercalcemia and hypercalcuria. [14]. These secondary systemic changes in the mineral metabolism axis make it impossible to assess the individual contribution of NaPi-IIa in the osteoclast solely on the basis of histology analysis.

Given the lack of conclusive evidence on the expression and role of NaPi-IIa in the osteoclast, we decided to i) study in detail the expression of sodium-dependent phosphate transporters during osteoclast differentiation, to ii) assess the impact of genetic loss of NaPi-IIa on osteoclast differentiation and resorption in vitro and to iii) study structural bone parameters by microcomputed tomography (μCT) analysis in NaPi-IIa WT and KO mice.

## Results

### Expression of sodium-dependent phosphate transporters during osteoclast differentiation

**Fig 1:**
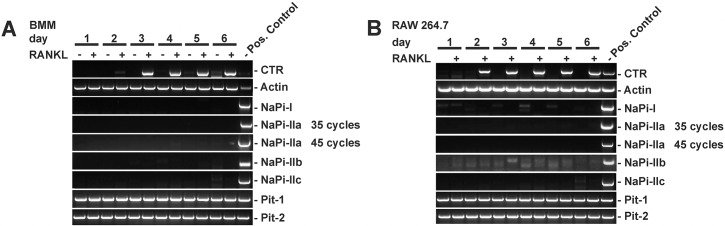
Expression of type I, II, and III phosphate transporter isoforms during osteoclast differentiation studied by endpoint RT-PCR. A) Primary bone-marrow-derived monocytes (BMMs) or B) RAW 264.7 cells were cultured with (+) or without (-) RANKL for 6 days. Total RNA was extracted and cDNA amplified by endpoint-RT-PCR using previously validated, specific primers for type I, type II (IIa, IIb, IIc) and type III (Pit-1, Pit-2) phosphate transporters. Calcitonin receptor (CTR) was used as a maker of differentiation to osteoclasts, β-actin was used as a positive RT-PCR control. Unless otherwise indicated, PCR was performed for 35 cycles. Positive controls were kidney cDNA with the exception of NaPi-IIb, where lung cDNA was used.

While several sodium-dependent phosphate transporters were found to be expressed in osteoclast precursors and mature osteoclasts, the time course of expression during RANKL (receptor activator of NF-кВ ligand)-mediated osteoclast differentiation has not been explored [8,10,12]. We first investigated mRNA expression of sodium-dependent phosphate transporters using endpoint RT-PCR with previously validated primers. Bone marrow-derived monocytes (BMMs) were cultured in the presence of 20 ng/ml RANKL and 30 ng/ml M-CSF or in the presence of vehicle (0.1% BSA) and 30 ng/ml M-CSF for a total of 6 days. As shown in Fig 1A, the osteoclast differentiation marker calcitonin receptor was specifically upregulated upon RANKL treatment and absent in vehicle treated cells. Using RT-PCR with previously described primers, we were unable to detect transcripts for NaPi-I, NaPi-IIa, IIb or IIc in vehicle or RANKL-treated BMMs, whereas the positive control PCRs yielded amplification products at the expected size. In contrast, type III phosphate transporters Pit-1 and Pit-2 were expressed in vehicle- and RANKL-treated cells. Similar results were obtained when we used RAW 264.7 cells, which were cultured in the presence of 50 ng/ml RANKL (Fig 1B).

**Fig 2:**
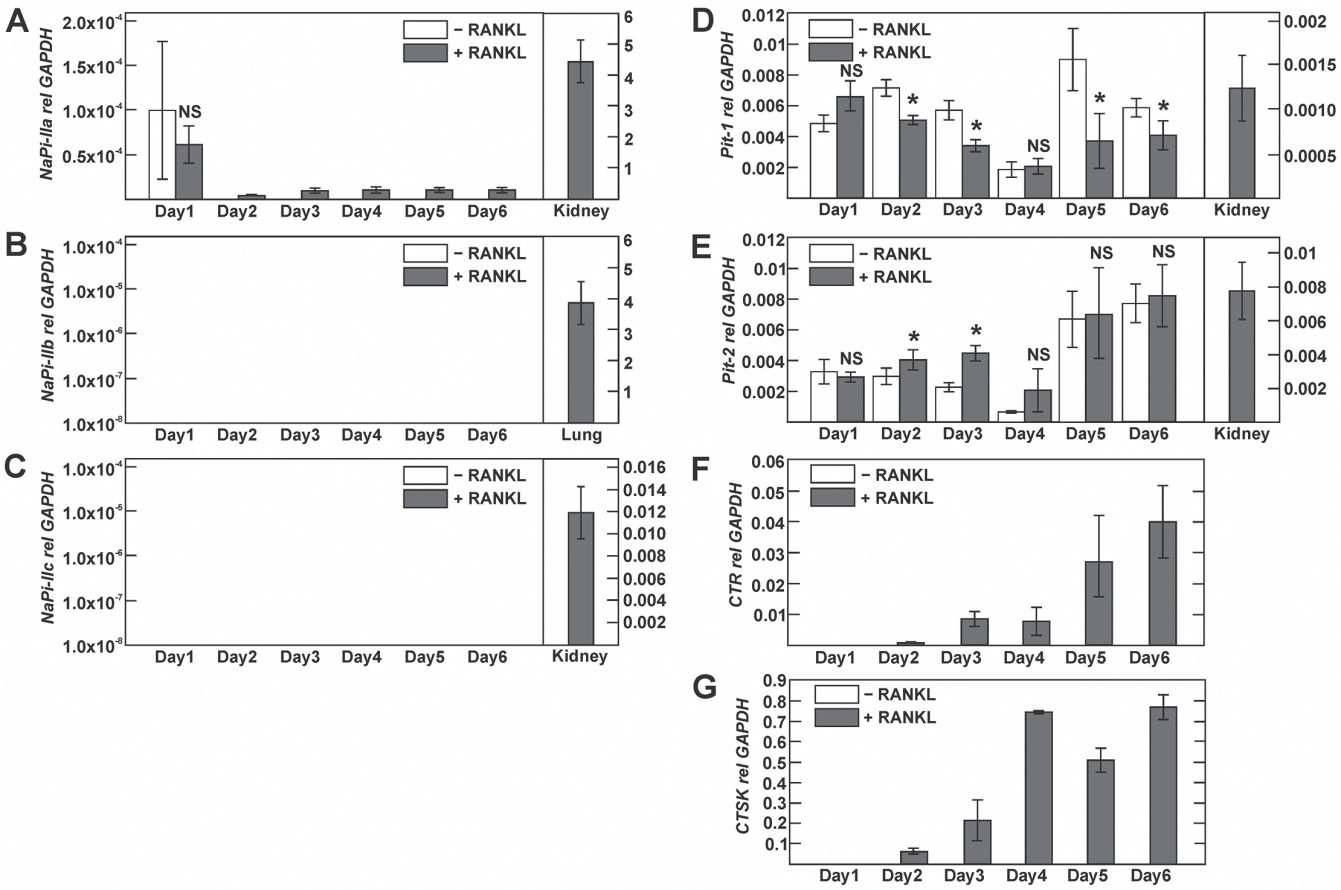
Expression of type I, II and III phosphate transporter isoforms during osteoclast differentiation of bone marrow-derived monocytes. Primary bone-marrow-derived monocytes (BMMs) were cultured with or without RANKL for 6 days. A-E) Phosphate transporter transcript expression was assessed by real-time PCR analysis using Taqman probes. F, G) Calcitonin receptor (CTR) and cathepsin K (CTSK) were used as markers of osteoclast differentiation. No signal was detected for NaPi-IIa in vehicle-treated BMMs at days 2–6 (A) and for NaPi-IIb and NaPi-IIc at any time point irrespective of the presence or absence of RANKL (not shown). Positive control PCRs in A-E were done with kidney cDNA for NaPi-IIa, NaPi-IIc, Pit-1 and Pit-2 and lung cDNA for NaPi-IIb. The amount of mRNA relative to GAPDH was calculated using the ΔCt method. Values are shown as means ±SD; n = 3 mice/group. * p <0.05; NS, not significant.

**Fig 3:**
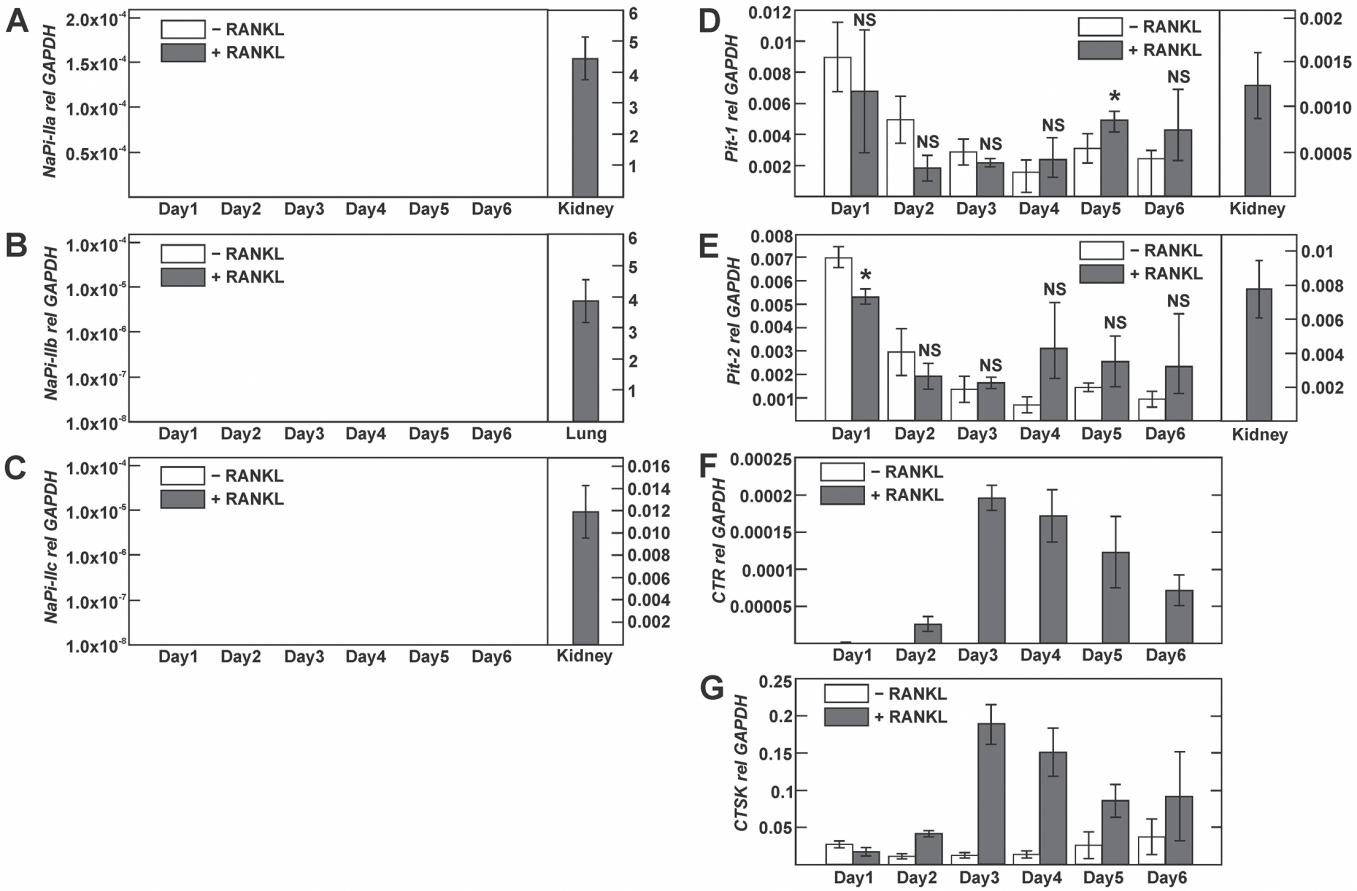
Expression of type II and III phosphate transporter isoforms during osteoclast differentiation of RAW 264.7 cells. RAW 264.7 cells were cultured with or without RANKL for 6 days. A-E) NaPi transcript expression was assessed by real-time PCR analysis. F, G) Calcitonin receptor (CTR) and cathepsin K (CTSK) were used as markers of osteoclast differentiation. No signal was detected for NaPi-IIa, NaPi-IIb and NaPi-IIc at any time point irrespective of the presence or absence of RANKL (not shown). Positive control PCRs in A-E were done with kidney cDNA for NaPi-IIa, NaPi-IIc, Pit-1 and Pit-2 and lung cDNA for NaPi-IIb. The amount of mRNA relative to GAPDH was calculated using the ΔCt method. Values are shown as means ±SD; n = 3 independent experiments/group. * p <0.05; NS, not significant.

We next employed taqman-based real-time PCR to quantify relative expression of type II and III phosphate transporters transcripts. Induction of osteoclast differentiation by RANKL was verified by concomitant expression analysis of the calcitonin receptor and cathepsin K transcripts (Figs. 2D and 2E and 3C and 3D). In cultured BMMs, we detected a weak signal for NaPi-IIa transcript, which declined beyond 1 day of culture and remained detectable thereafter only in RANKL-stimulated but not vehicle-treated BMMs (Fig 2A). However, we could not detect NaPi-IIa transcript in vehicle or RANKL-treated RAW 264.7 cells (Fig 3A). Similarly, we were unable to detect NaPi-IIb or—IIc transcripts, both in RAW 264.7 and cultured BMMs (Figs 2B and 2C and 3B and 3C), while significant transcript expression was detected for these NaPi-II isoforms in kidney (NaPi-IIa,-IIc) or lung (NaPi-IIb) (Fig 2B and 2C). By contrast, type III phosphate transporters Pit-1 and Pit-2 transcripts were present in RAW 264.7- and BMM-derived osteoclasts, but the presence or absence of RANKL had little influence on their expression levels (Figs 2D and 2E and 3D and 3E).

**Fig 4:**
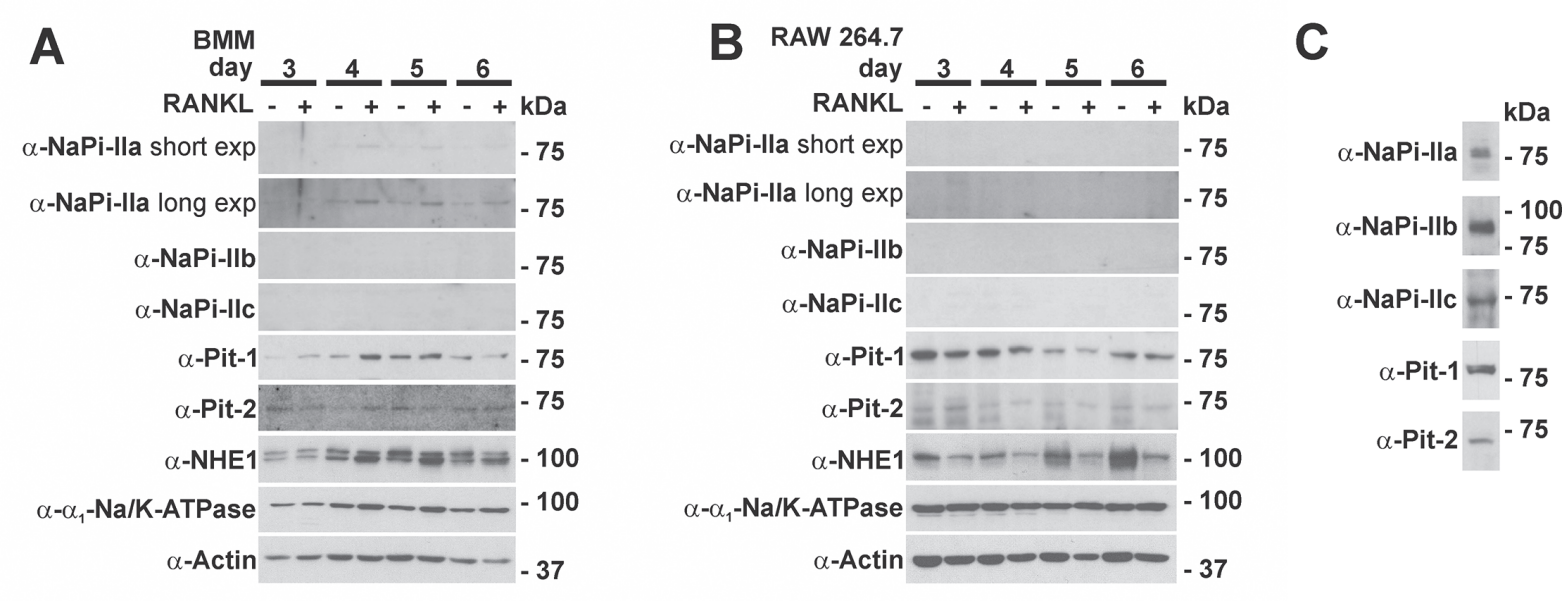
Western Blot analysis of type II and III phosphate transporters during osteoclast differentiation. A) BMMs or B) RAW 264.7 cells were cultured with or without RANKL for a total of 6 days. At day 3 to 6 cell lysates were prepared and equal amounts of protein (50 μg) were separated by SDS-PAGE and probed with indicated antibodies. In contrast to BMM cultures (A), no NaPi-IIa signal was detectable in the RAW 264.7 cell cultures (B). No NaPi-IIb and NaPi-IIc signal was detected in both cell types irrespective of the presence or absence of RANKL. Both Pit-1 and Pit-2 are expressed during osteoclast differentiation. (C) Validation of antibodies using kidney lysate for detection of NaPi-IIa, NaPi-IIc, Pit1 and Pit2 and ileum mucosa for NaPi-IIb. Blots are representative of two independent experiments. NHE1: sodium/proton exchanger 1.

We next analyzed the expression of the different SLC34 phosphate transporters on protein level during osteoclast differentiation using previously validated antibodies [16–18]. As shown in Fig 4, we detected a weak NaPi-IIa signal in the BMM-derived osteoclasts, but not in the RAW 264.7 derived osteoclasts. Consistent with mRNA expression analysis, we could not detect NaPi-IIb or—IIc protein by Western blot neither in osteoclasts nor in osteoclast precursors, whereas controls Western blots yielded bands at the expected size in ileum and kidney lysates, respectively (Fig 4C). [19,20].

These data indicate that of the type II phosphate transporters only NaPi-IIa is present at low levels in BMM- but not in Raw 264.7-derived osteoclasts.

### Differentiation and resorptive activity of NaPi-IIa-deficient osteoclasts

**Fig 5:**
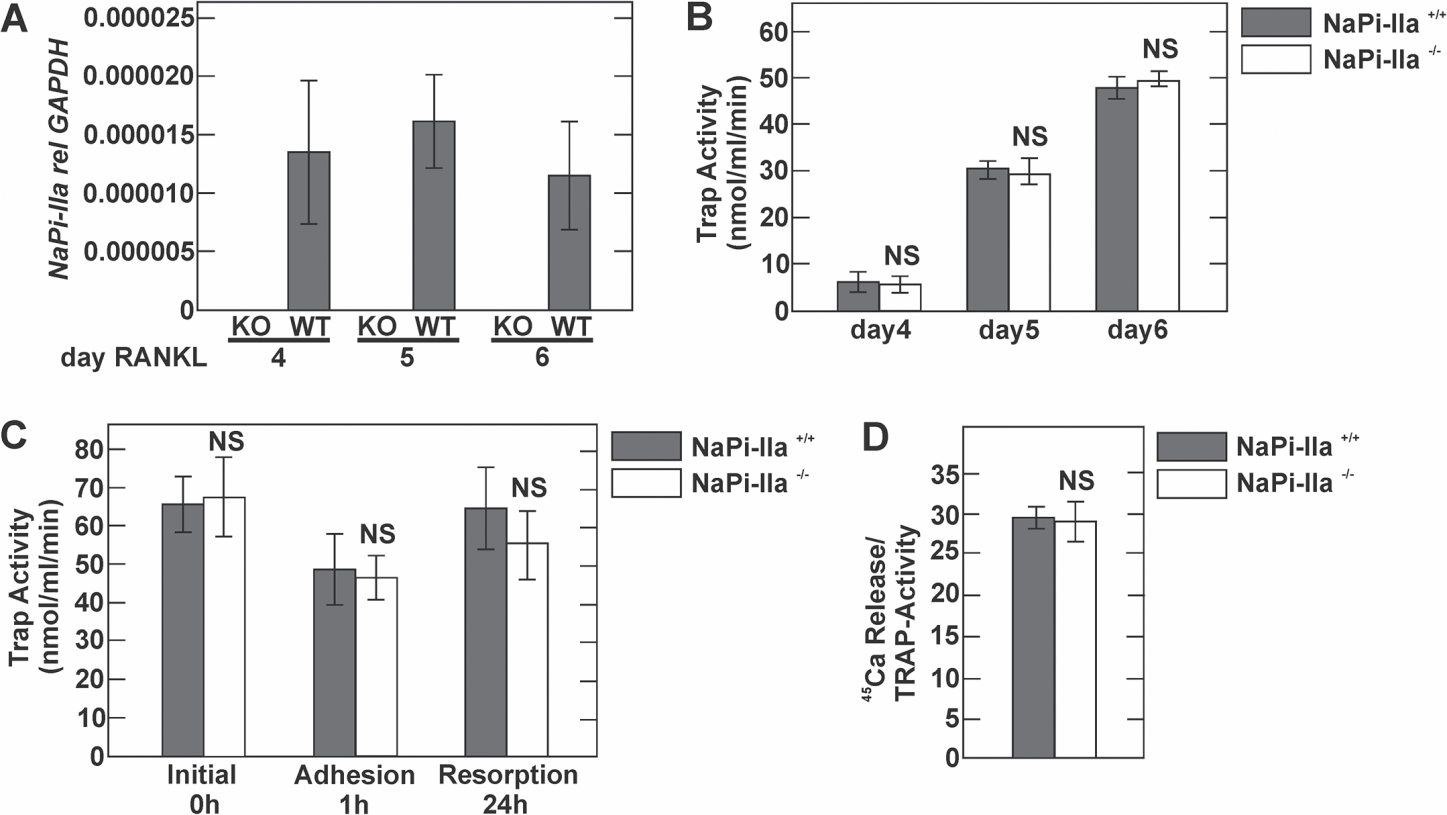
In vitro characterization of NaPi-IIa deficient-osteoclasts. BMMs isolated from NaPi-IIa WT and KO mice were cultured with RANKL for 4, 5 or 6 days. A) NaPi-IIa transcript expression assessed by real-time PCR analysis at days 4, 5 or 6 of RANKL stimulation. Note the absence of NaPi-IIa transcript in osteoclasts derived from NaPi-IIa KO mice. B) TRAP activity of osteoclast cultures at days 4, 5 and 6. C) TRAP activity of osteoclast cultures harvested after 0, 1 and 24h after reseeding on calcium-phosphate coated plates. D) Resorptive activity of osteoclast cultures quantified as ^45^Ca content in the supernatant compared to TRAP activity.

Previous reports suggested that NaPi-IIa may play an important role in osteoclast-mediated bone resorption [8–10]. However, direct experimental evidence supporting this notion is lacking. To test for a possible role of NaPi-IIa in osteoclasts, we characterized differentiation as well as resorptive activity of osteoclasts that lack NaPi-IIa *in vitro*. For this purpose, BMMs were isolated from NaPi-IIa WT and KO mice and osteoclast differentiation was induced by addition of 20 ng/ml RANKL and 30 ng/ml M-CSF. At days 4, 5 and 6 of stimulation, cells were harvested and NaPi-IIa transcript as well as tartrate-resistant acid phosphatase (TRAP) activity quantified. As shown in Fig 5A, NaPi-IIa transcript levels were undetectable in osteoclasts derived from KO mice whereas low NaPi-IIa mRNA expression was detectable in WT osteoclasts at all time points. However, osteoclast differentiation, as determined by TRAP activity, was not different between the two groups at 4, 5 or 6 days of RANKL treatment (Fig 5B).

The resorptive activity of osteoclasts was determined according to [21] with a few modifications [22]. Osteoclasts were collected at day 5 of differentiation and seeded into calcium-phosphate (CaP) coated wells, containing ^45^Ca as a tracer. Resorption was calculated as ^45^Ca released into the cell culture supernatant normalized to TRAP activity at 24h. To determine survival/ development of osteoclast lineage cells, TRAP activity was determined immediately after seeding and after 1 and 24h of culture on the CaP substrate. As shown in Fig 5C, TRAP activity was not different between osteoclasts derived from BMMs of NaPi-IIa WT and NaPi-IIa KO mice. Also, resorptive activity was not different between NaPi-IIa WT and KO osteoclasts (Fig 5D).

### Structural bone parameters of NaPi-IIa KO mice

**Fig 6:**
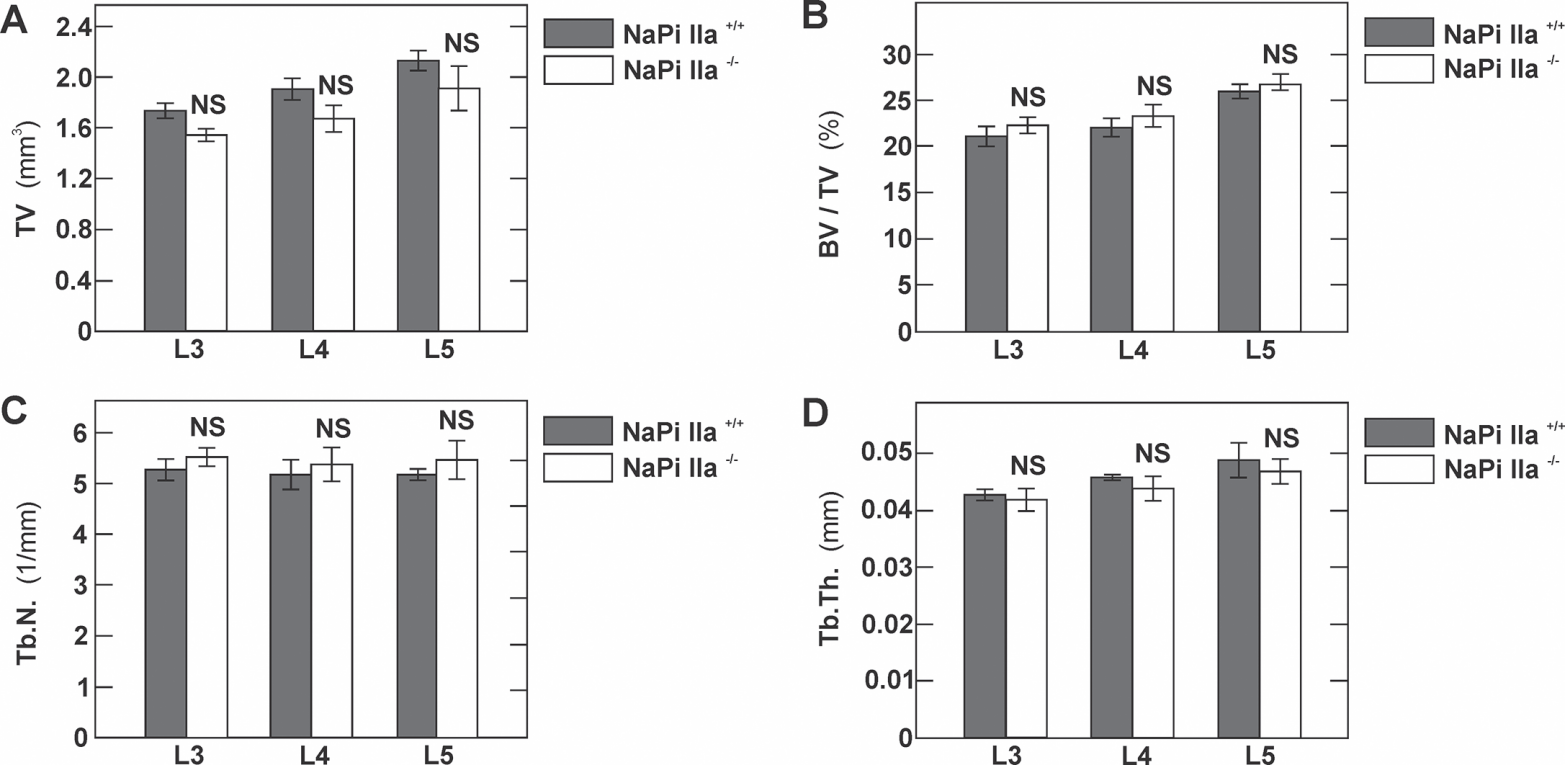
Structural bone parameters of NaPi-IIa KO mice. Lumbar vertebrae (# 3, 4 and 5) of 12 weeks old NaPi-IIa WT and KO mice were isolated and analyzed by μCT. Histograms representing structural parameters of the lumbar vertebrae: Total volume, bone volume fraction (BV/TV), trabecular number (Tb.N.) and trabecular thickness (Tb.Th.). Data are shown as mean ± SD, n = 4 mice in each group. NS, not significant.

To determine the impact of genetic NaPi-IIa deficiency on bone mass and on structural bone parameters, we performed high-resolution micro-computed tomography studies of lumbar vertebrae of 10 weeks old NaPi-IIa WT and KO mice. As depicted in Fig 6, and in line with previous histomorphometric analysis of same aged mice [15], we did not detect any differences in structural bone parameters between the two groups of mice.

## Discussion

In this study, we i) systematically characterized the expression profiles of sodium-dependent phosphate transporters during osteoclast differentiation and ii) assessed the consequences of NaPi-IIa ablation on osteoclast differentiation and resorption. Our data indicate that NaPi-IIa is present in freshly isolated BMMs and in BMM-derived osteoclasts. Interestingly, time course experiments revealed that expression levels of NaPi-IIa are the highest in freshly isolated BMMs. Starting on day 2 of culture, NaPi-IIa expression was only detectable in RANKL-treated cells but not in vehicle-treated cells. Western blot analysis indicated the same pattern of NaPi-IIa expression on protein level. Unlike previously reported, we were unable to detect NaPi-IIa on RNA or protein level in RANKL- or vehicle-stimulated RAW 264.7 cells. The reason for this apparent discrepancy to the work of Gupta et al. [9] is unclear but may involve different culture conditions or sources of reagents employed in the current study.

A role for NaPi-IIa in osteoclast function has long been suspected [8–10,12,13]. Although low, the differential expression of NaPi-IIa upon RANKL stimulation we observed supported this notion. Conversely, our experiments show that NaPi-IIa is dispensable i) for osteoclast differentiation and ii) for osteoclast-mediated resorption in vitro.

Unlike NaPi-IIa, we were unable to detect the two other SLC34 isoforms, NaPi-IIb and—IIc by RT-PCR, real-time PCR or Western blotting at any time point during osteoclast differentiation. At first glance, this may not be surprising, given the known restricted expression of NaPi-IIb to intestine, liver, lung, testis, and NaPi-IIc to the kidney [23,24]. Interestingly, however, recent data challenged the view that NaPi-IIc is a kidney-specific phosphate transporter. While full body KO NaPi-IIc mice exhibit hypercalcemia, hypercalciuria and increased 1,25-OH vitamin D_3_ levels, kidney-specific deletion of NaPi-IIc does not cause any alterations in mineral metabolism, strongly suggesting an extrarenal role of NaPi-IIc in intestine and/or bone [17,25]. Additional studies with osteoblasts and osteocytes will be required to definitively assess the role of SLC34 transporters in the bone.

In addition to NaPi-IIa, both type III phosphate can be detected on RNA and protein level in osteoclasts but with little variation of expression upon RANKL compared to vehicle treatment. On a functional level, Ito et al. identified significant sodium-dependent phosphate transport in vehicle-stimulated RAW 264.7 cells that changed only little upon RANKL stimulation [13]. These functional data are in agreement with the findings of our present study that revealed little variation in expression levels of the type III phosphate transporters during osteoclast differentiation. Based on these functional data and the much lower RNA expression levels of NaPi-IIa, type III phosphate transportersPit-1 and Pit-2 likely mediate the main sodium-driven phosphate transport in osteoclasts and osteoclast precursors. Whether sodium-driven phosphate transport in osteoclasts is indeed entirely mediated by the combined action of Pit-1 and Pit-2 and what are the roles of these 2 transporters in the osteoclast, remains to be determined.

Autosomal-dominant mutations in Pit-2 were recently shown to cause familial idiopathic basal ganglia calcification (FIBCG) [26]. No abnormalities in serum electrolytes or calciotropic hormones and no obvious bone phenotype have been reported from subjects with FIBCG. Pit-2 KO mice recapitulate the human disease in that they exhibit basal ganglia calcification, but a bone phenotype of these mice has not been reported [27]. In contrast to Pit-2, complete Pit-1 KO is embryonically lethal in mice [28]. Mice with two hypomorphic Pit-1 alleles and 85% reduced Pit-1 expression exhibit growth retardation, liver abnormalities and anemia, but no defects in skeleton formation and normal bone mineralization, up to 300 days of age [28,29]. In addition, Pit-1 hypomorphic mice exhibited upregulation of Pit-2 in the bone, suggesting possible functional redundancy [29]. Strikingly, Pit-1 appears to further exert a phosphate transport-independent role (which is not shared by Pit-2), regulating p38 mitogen-activated protein (MAP) kinase activation and cell proliferation [30]. Given the almost ubiquitous presence of Pits, the embryonic lethality of full body Pit-1 KO and the possible functional overlaps, only selective and combined osteoclast-specific deletions, similar to what was performed in vascular smooth muscle cells [31,32], will allow a definitive statement on the physiological role of type III phosphate transporters in the osteoclast.

In osteoclast precursors, proton-driven and sodium-driven phosphate transport activities are of similar magnitude [13]. During RANKL stimulation, however, the proton-driven transport activity is strongly upregulated. As such, a large fraction of phosphate transport in mature osteoclasts is proton-driven [13]. With regard to the mandatory low pH in the hemivacuole for bone degradation, it energetically makes sense to couple phosphate with proton transport. Vectorial transcellular phosphate transport in polarized cells requires phosphate efflux systems in addition to phosphate influx systems. Phosphate efflux transport has been functionally described in osteoclasts in great detail, but the molecular identity of the transporter(s) is yet to be elucidated [13]. A possible candidate for phosphate efflux is XPR1, which was strongly upregulated during RANKL-induced osteoclast differentiation [33]. XPR1 is a multipass membrane protein and was originally identified as the receptor for xenotropic- and polytropic murine leukemia retroviruses [34]. Overexpression of XPR1 induces phosphate efflux, and knock-down reduces phosphate efflux in mammalian cells [35]. However, if XPR1 mediates sodium-independent phosphate efflux directly or just indirectly, remains unknown. In addition, its physiological function in the osteoclast needs to be established. Although an ultimate proof for the concept is lacking, the differential expression of various sodium-dependent and-independent phosphate transport systems in osteoclasts strongly suggests that a vectorial, transcellular phosphate transport route is present in bone resorbing osteoclasts.

In summary, our data demonstrate that NaPi-IIa is the only SLC34 member expressed during RANKL-induced osteoclast differentiation but is dispensable for osteoclast differentiation and bone resorption. However, type III phosphate transporters exhibit significant expression throughout osteoclast differentiation, indicating that the SLC20 family members Pit-1 and Pit-2 are the main transporters responsible for sodium-driven phosphate uptake in osteoclasts. The functional relevance of Pit transporters in osteoclasts remains unknown at the moment and needs to be addressed in future studies.

## Materials and Methods

Unless specified otherwise, all chemicals and reagents were obtained from Sigma. Statistical analysis was done using Student´s t test. All statistical tests were two-sided and a p value <0.05 was considered statistically significant.

### Cell culture and osteoclast differentiation

The murine macrophage / monocyte cell line RAW 264.7 was obtained from ATCC. Cells were maintained in α-MEM (Invitrogen, Carlsbad, CA), supplemented with 10% FBS (Invitrogen), 100 U/ml penicillin and 100 U/ml streptomycin, and grown in a humidified 95%/5% air/CO_2_ atmosphere incubator at 37ºC. To induce osteoclast differentiation, cells were cultured in the presence of 50 ng/ml soluble RANKL (PeproTech, Hamburg, Germany) for indicated time points with changes of medium and RANKL every other day. Mouse bone marrow cells were obtained from femora and tibiae of 8–12 week old mice. Bones were dissected and bone marrow cells flushed out with Hanks Balanced Salt Solution supplemented with 100 U/ml penicillin and 100 U/ml streptomycin. Cells were then sedimented, resuspended, counted and plated in α-MEM supplemented with 10% FBS in the presence of 30 ng/ml M-CSF (Chiron, Emeryville, CA) and 20 ng/ml RANKL with medium changes every 3 days.

### RNA isolation, RT-PCR and quantitative real-time PCR

Total RNA was isolated using Trizol reagent as detailed in the manufacturer’s protocol (Invitrogen, Carlsbad, Ca). Reverse transcription was performed using the Taqman Reverse Transcription kit (Life Technologies/ABI, Rotkreuz, CH). PCR was performed as described previously [36]. For end-point PCR, primers overlapping two exons were used to avoid artifacts due to genomic DNA contamination. The following, previously described primers were used: NaPi-I FW: 5’-AAG AAA GTT CCA GGG TTC TG-3’, NaPi-I RV: 5’- AAG GGA GGT GCC CAT TTG AC-3’ [12]; NaPi-IIa FW: 5’-TTT ATC CAG TAC TTC CCG AGC AGG-3’; NaPi-IIa RV: 5’-CCC GAG ATG TTG AAG AAG AAG TG-3’ [8]; NaPi-IIb FW: 5’-GAC CTG CCT GAA CTC CAG-3’; NaPi-IIb RV: 5’-ATC GTG TTG GTG ATG GAG G-3’ [37]; NaPi-IIc FW: 5’-CTG GTG GAC CGG AGT CTG-3’; NaPi-IIc RV: 5’-GGA CAG TCA GCA ACT TAG AGG-3’[37]; Pit-1 FW: 5’-CCC ATG GAC CTG AAG GAG GAG-3’ and Pit-1 RV: 5’-CCA TGG GCA AAT GAC CCA AAG-3’[12]; Pit-2 FW: 5’-CGT GTG GCT ATT CGT GTG TCC-3’and Pit-2 RV: 5’-TCT ACG TGG ATT TTG TGC AGC-3’ [12]; Calcitonin receptor FW: 5’-TTC AAG AAC CTT AGC TGC CAG AG-3’ and Calcitonin receptor RV: 5’-CTG GAA ATG AAT CAG AGA GTG C-3’ [12]; β-actin FW: 5’- AAC CGT GAA AAG ATG ACC CAG-3’ and β-actin RV: 5’- CCA TCT CCT GCT CGA AGT C-3’ [8].

Realtime PCR was performed using pre-synthesized Taqman-based Assays-on-Demand (Life Technologies/ABI, Rotkreuz, CH) on an ABI ViiA 7 System. The following AoDs were employed: NaPi-IIa (Mm00441450_m1), NaPi-IIb (Mm01215846_m1), NaPi-IIc (Mm00551746_m1), Pit-1 (Mm00489378_m1), Pit-2 (Mm00660203_m1), Calcitonin receptor (Mm00432282_m1), Cathepsin K (Mm00484039_m1), GAPDH (Mm99999915_g1). Ct values for triplicate technical replicates were averaged and the amount of mRNA relative to GAPDH was calculated using the ΔCt method.

### Antibodies and immunoblotting

Antibodies used in the study were from the following sources: monoclonal anti-NHE1 and monoclonal anti-α_1_ subunit of the Na/K-ATPase (Millipore, Billerica, MA), polyclonal anti-actin (Santa Cruz Biotechnology, Santa Cruz, CA). Polyclonal rabbit anti-NaPi-IIa, anti-NaPi-IIb, anti-NaPi-IIc, anti-Pit-1 and anti-Pit-2 were characterized and validated previously [16–18]. Immunoblotting was performed as described [36].

### In vitro osteoclast differentiation and resorption assay

To assess the differentiation of osteoclasts *in vitro*, bone marrow cells were seeded in 96 well plates (30’000 cells/well in 100 μl) for determination of TRAP activity in cell lysates. Osteoclast differentiation was induced by supplementing the culture medium with M-CSF (30 ng/ml) and RANKL (20 ng/ml, respectively). TRAP determination was performed as described previously [38,39]. The resorptive activity of osteoclasts was determined according to [21] with a few modifications [22]. For this purpose, osteoclasts were grown from M-CSF-dependent non-adherent osteoclast progenitors on up-cell dishes (Thermo Scientific, Waltham, MA, USA) and collected at day 5 of differentiation. The cells were seeded into CaP coated wells, containing ^45^Ca as a tracer. To produce the crystalline CaP layer, equal amounts of 0.12 M Na2HPO4 and 0.2 M CaCl2 (in 50 mM Tris/ HCl, pH 7.4) were incubated at 37°C/ 5% CO2 overnight. Upon mixing, the newly formed slurry of CaP was washed twice in sterile water and subsequently resuspended in 1 ml of sterile water per 100 μl of the slurry. ^45^Ca was added at 1.2 MBeq/ 10 ml as CaCl2, and 100 μl of the suspension was added to wells of 48-well plates. The wells were dried in a laminar flow and sterilized by UV irradiation. The release of ^45^Ca into the culture supernatants during 24h was used as a parameter to assess the resorptive activity of the cells. To determine survival/ development of osteoclast lineage cells, TRAP activity was determined immediately after seeding and after 1 and 24h of culture on the CaP substrate. Resorption was calculated as ^45^Ca released into the cell culture supernatant normalized to TRAP activity at 24h.

### Mice

All animal experiments were approved Veterinäramt of the Kanton Bern, Switzerland. Mice had free access to water and standard chow diet (#3436 from Provimi Kliba AG, Kaiseraugst, Switzerland) and were maintained on a 12 hours light/12 hours dark cycle. Generation and characterization of mice with a targeted disruption of the *NaPi-IIa* gene were reported in detail previously [14]. Genotyping was performed as described [14]. For experiments, only male littermates were used. All experiments were performed on littermates with a pure C56BL/6J background bred from heterozygous mice.

### Microcomputed CT analysis of bone

Bone structure was determined in a μCT40 System (Scanco, Brüttisellen, Switzerland). Freshly excised bone specimen were fixed in 4% paraformaldehyde, rinsed overnight with tap water and immersed in 70% ethanol. MicroCT scans were performed in 70% ethanol at the highest resolution, with a voxel size of 8 μm [40].
